# Higher red blood cell distribution width is associated with a worse virologic and clinical situation in HIV-infected patients

**DOI:** 10.1186/1758-2652-13-S4-P69

**Published:** 2010-11-08

**Authors:** S Puerta, M Gallego, R Palacios, J Ruiz, E Nuño, M Márquez, J Santos

**Affiliations:** 1 Hosp. Virgen de la Victoria, UGC Enfermedades Infecciosas, Malaga, Spain

## Background

A high level of red blood cell distribution width (RDW) is a novel prognostic marker that may reflect an underlying inflammatory state. It has recently shown that when increased, it is related to cardiovascular disease, mortality, and metabolic syndrome (MetS) in the general population.

## Objectives

To analyse the potential relation between high levels of RDW and cardiovascular risk (CVR) and MetS in HIV-patients.

## Patients and methods

Observational, cross-sectional study of a series of HIV-outpatients attended in our Hospital. Demographic, anthropometric, clinical, and fasting lab data were recorded in all cases. CVR at 10 years was evaluated by Framingham equation, and MetS diagnosed according to the National Cholesterol Education Program criteria. Statistic program: SPSS 17.0.

## Results

666 patients were included, 79.3% were men, and mean age was 44.7 years. Mean CD4 count was 506 cells/mm^3^, 87.5% of the patients were on antiretroviral therapy, and 85.3% had undetectable HIV viral load. Mean RDW was 13.07% (range: 7.7-33.6%; 75th percentile 14,1%), with a prevalence of MetS of 15.7, 9.3, 18.8 and 16.6% first through fourth RDW quartile, and of patients with CVR >20% of 8.4, 4.0, 4.4 and 6.4%, respectively (p>0,05). The highest quartile of RDW (>14.1%) was associated with AIDS (OR 1.6, 95%CI 1.0-2.4; p 0.02), detectable HIV viral load (OR 1.5, 95%CI 1.01-2.4; p 0.04), and hypertension (OR 2.3, 95%CI 1.4-4.0; p 0.001)*.*

## Conclusions

In HIV-infected outpatients, higher RDW is related with detectable HIV viral load and with AIDS. Although it was associated with a traditional CVR factor as hypertension, we found no relation with MetS nor with higher CVR.
